# Whole-Genome Shotgun Sequence of *Escherichia coli* Strain MN067 from India, a Commensal Bacterium with Potent Pathogenic Ability

**DOI:** 10.1128/genomeA.00054-17

**Published:** 2017-03-23

**Authors:** Daram Nagarjuna, Rajni Gaind, Rakesh Singh Dhanda, Manisha Yadav

**Affiliations:** a Dr. B. R. Ambedkar Center for Biomedical Research, University of Delhi, Delhi, India; b Department of Microbiology, Vardhman Mahavir Medical College (VMMC) and Safdarjung Hospital, Delhi, India; c Department of Translational and Regenerative Medicine, Postgraduate Institute of Medical Education and Research (PGIMER), Chandigarh, India

## Abstract

*Escherichia coli* is one of the most frequently prevalent pathogens, causing infections in health care settings throughout the world. Here, we report the whole-genome sequence of MN067, a commensal bacterium with a pathogenic potential.

## GENOME ANNOUNCEMENT

Health care–associated infections are a major public health threat throughout the world, but they are often preventable. Patients admitted to intensive care units (ICUs) are at a higher risk of developing bacteremia and septicemia due to invasive procedures such as peripheral cannulation, central venous catheter placement, tracheal incubation, and ventilation (1). The human gut microbiota has long been known to contribute to both health and disease (2). Although *Escherichia coli* from the gut is a well-established commensal bacterium, it has the potential to cause disease. *E. coli* is among the most frequently prevalent pathogens causing infection in ICUs (3). However, the propensity of disease is directly affected by both host and bacterial factors, among which susceptibility of the host is crucial (4). Here, we report the whole-genome shotgun sequence of *E. coli* MN067, a gut isolate from a patient admitted to the ICU of Safdarjung Hospital, New Delhi, India. The genomic content of MN067 was analyzed for potential genes that may be involved in virulence and antibiotic resistance.

Whole-genome sequencing was performed using the Illumina MiSeq platform with 2 × 300-bp chemistry using paired orientation, which produced 0.87 Gb of high-quality data, with 1,857,915 reads. The generated reads were quality-filtered using Trimmomatic version 0.35 and adapter trimming. Sequencing assembly was performed using SPAdes with optimized *k*-mer sizes, followed by SSPACE for scaffolding, which resulted in a draft genome of 5.6 Mb with 225 scaffolds, a G+C content of 50.3%, an *N*_50_ of 109,126 bp, 5,651 coding sequences and 99 RNAs. After assembly, gene prediction was performed using Prodigal. As a result, a total number of 5,217 genes were predicted with an average gene size of 935 bp. Using BLASTx, 5,214 genes out of a total of 5,217 found specific hits, while three did not find any specific hit against the NR database during functional annotation. GO annotation was performed using the paid version of Blast2GO PRO. A total of 3,983 genes were annotated in various functional categories, including biological process (3,561 genes), cellular component (2,574 genes), and molecular function (3,345 genes). Pathway analysis was performed using Blast2GO PRO-KEGG.

The genome of MN067 was analyzed by PathogenFinder (5) and was established as a human pathogen. The *in silico* multilocus sequence type analysis showed that MN067 belongs to ST10 (6). Analysis by ResFinder version 2.1 showed that MN067 lodged 10 genes (data not shown) that confer resistance to known major antimicrobials like beta lactams, fluoroquinolone, aminoglycosides (7). The peculiar pathogenicity potential of the MN067 genome can be characterized further in future studies through comparisons with existing *E. coli* genomes. A comparative genomics analysis of this strain with other closely related commensal bacteria will further our understanding about the distinct features of this pathogenic commensal bacterium.

### Accession number(s).

This whole-genome shotgun project has been deposited at DDBJ/ENA/GenBank under the accession number MKQI00000000. The version described in this paper is the first version, MKQI01000000.
